# Supplementary material for: HIVprotI: an integrated web based platform for prediction and design of HIV proteins inhibitors
Source: J Cheminform. 2018 Mar 9;10:12. doi: 10.1186/s13321-018-0266-y (PMC5845081; doi:10.1186/s13321-018-0266-y)
Supplement: Supplementary file 2 — Additional file 2. Source code of HIVProtI web server. [file 13321_2018_266_MOESM2_ESM.zip › HIVprotI_Source-code/hivprotI/weka-3-6-6/doc/weka/associations/tertius/LiteralSet.html]

LiteralSet


---


|  |  |  |
| --- | --- | --- |
| **Overview** \| **Package** \| **Class** \| **Tree** \| **Deprecated** \| **Index** \| **Help** \| **Weka's home** | |  |
| **PREV CLASS**   **NEXT CLASS** | **FRAMES**    **NO FRAMES**     **All Classes** |  |
| SUMMARY: NESTED \| FIELD \| CONSTR \| METHOD | DETAIL: FIELD \| CONSTR \| METHOD |  |


---


## weka.associations.tertius Class LiteralSet

```
java.lang.Object
  weka.associations.tertius.LiteralSet
```

**All Implemented Interfaces:**: java.io.Serializable, java.lang.Cloneable, RevisionHandler

**Direct Known Subclasses:**: Body, Head

---

``` public abstract class LiteralSet extends java.lang.Object implements java.io.Serializable, java.lang.Cloneable, RevisionHandler ```

Class representing a set of literals, being either the body or the head
of a rule.

**Version:**
:   $Revision: 1.7 $

**Author:**
:   Amelie Deltour

**See Also:**: Serialized Form

---

**Constructor Summary**

`LiteralSet()`
  
          Constructor for a set that does not store its counter-instances.

`LiteralSet(Instances instances)`
  
          Constructor initializing the set of counter-instances to all the instances.


| **Method Summary** | |
| --- | --- |
| `void` | `addElement(Literal element)`             Add a Literal to this set. |
| `abstract  boolean` | `canKeep(Instance instance, Literal newLit)`             Test if an instance can be kept as a counter-instance, given a new literal. |
| `java.lang.Object` | `clone()`             Returns a shallow copy of this set. |
| `boolean` | `contains(Literal lit)`             Test if this LiteralSet contains a given Literal. |
| `boolean` | `counterInstance(Instance instance)`             Test if an instance is a counter-instance of this LiteralSet. |
| `boolean` | `counterInstance(Instance individual, Instance part)`             Test if an individual instance, given a part instance of this individual, is a counter-instance of this LiteralSet. |
| `java.util.Iterator` | `enumerateLiterals()`             Enumerate the literals contained in this set. |
| `double` | `getCounterInstancesFrequency()`             Get the frequency of counter-instances of this LiteralSet in the data. |
| `int` | `getCounterInstancesNumber()`             Get the number of counter-instances of this LiteralSet. |
| `Literal` | `getLastLiteral()`             Give the last literal added to this set. |
| `int` | `getType()`             Give the type of properties in this set (individual or part properties). |
| `boolean` | `hasMaxCounterInstances()`             Test if all the intances are counter-instances. |
| `boolean` | `isEmpty()`             Test if this set is empty. |
| `abstract  boolean` | `isIncludedIn(Rule otherRule)`             Test if this LiteralSet is included in a rule. |
| `boolean` | `negationIncludedIn(LiteralSet otherSet)`             Test if the negation of this LiteralSet is included in another LiteralSet. |
| `int` | `numLiterals()`             Give the number of literals in this set. |
| `boolean` | `overFrequencyThreshold(double minFrequency)`             Test if this LiteralSet has more counter-instances than the threshold. |
| `abstract  java.lang.String` | `toString()`             Gives a String representation for this set of literals. |
| `void` | `upDate(Instances instances)`             Update the number of counter-instances of this set in the dataset. |

**Methods inherited from class java.lang.Object**

`equals, getClass, hashCode, notify, notifyAll, wait, wait, wait`

**Methods inherited from interface weka.core.RevisionHandler**

`getRevision`

**Constructor Detail**

### LiteralSet

```
public LiteralSet()
```

:   Constructor for a set that does not store its counter-instances.

---


### LiteralSet

```
public LiteralSet(Instances instances)
```

:   Constructor initializing the set of counter-instances to all the instances.

    **Parameters:**: `instances` - The dataset.


**Method Detail**

### clone

```
public java.lang.Object clone()
```

:   Returns a shallow copy of this set.
    The structured is copied but the literals themselves are not copied.

    :   **Overrides:**: `clone` in class `java.lang.Object`
    :   **Returns:**: A copy of this LiteralSet.

---


### upDate

```
public void upDate(Instances instances)
```

:   Update the number of counter-instances of this set in the dataset.
    This method should be used is the set does not store its counter-instances.

    :   **Parameters:**: `instances` - The dataset.

---


### getCounterInstancesNumber

```
public int getCounterInstancesNumber()
```

:   Get the number of counter-instances of this LiteralSet.

    :   **Returns:**: The number of counter-instances.

---


### getCounterInstancesFrequency

```
public double getCounterInstancesFrequency()
```

:   Get the frequency of counter-instances of this LiteralSet in the data.

    :   **Returns:**: The frequency of counter-instances.

---


### overFrequencyThreshold

```
public boolean overFrequencyThreshold(double minFrequency)
```

:   Test if this LiteralSet has more counter-instances than the threshold.

    :   **Parameters:**: `minFrequency` - The frequency threshold. **Returns:**: True if there are more counter-instances than the threshold.

---


### hasMaxCounterInstances

```
public boolean hasMaxCounterInstances()
```

:   Test if all the intances are counter-instances.

    :   **Returns:**: True if all the instances are counter-instances.

---


### addElement

```
public void addElement(Literal element)
```

:   Add a Literal to this set.

    :   **Parameters:**: `element` - The element to add.

---


### isEmpty

```
public final boolean isEmpty()
```

:   Test if this set is empty.

    :   **Returns:**: True if the set is empty.

---


### numLiterals

```
public final int numLiterals()
```

:   Give the number of literals in this set.

    :   **Returns:**: The number of literals.

---


### enumerateLiterals

```
public final java.util.Iterator enumerateLiterals()
```

:   Enumerate the literals contained in this set.

    :   **Returns:**: An Iterator for the literals.

---


### getLastLiteral

```
public Literal getLastLiteral()
```

:   Give the last literal added to this set.

    :   **Returns:**: The last literal added.

---


### negationIncludedIn

```
public boolean negationIncludedIn(LiteralSet otherSet)
```

:   Test if the negation of this LiteralSet is included in another LiteralSet.

    :   **Parameters:**: `otherSet` - The other LiteralSet. **Returns:**: True if the negation of this LiteralSet is included in the other LiteralSet.

---


### contains

```
public boolean contains(Literal lit)
```

:   Test if this LiteralSet contains a given Literal.

    :   **Parameters:**: `lit` - The literal that is looked for. **Returns:**: True if this literal is contained in this LiteralSet.

---


### getType

```
public int getType()
```

:   Give the type of properties in this set (individual or part properties).

---


### counterInstance

```
public boolean counterInstance(Instance individual,
                               Instance part)
```

:   Test if an individual instance, given a part instance of this individual,
    is a counter-instance of this LiteralSet.

    :   **Parameters:**: `individual` - The individual instance.: `part` - The part instance. **Returns:**: True if the individual is a counter-instance.

---


### counterInstance

```
public boolean counterInstance(Instance instance)
```

:   Test if an instance is a counter-instance of this LiteralSet.

    :   **Parameters:**: `instance` - The instance to test. **Returns:**: True if the instance is a counter-instance.

---


### canKeep

```
public abstract boolean canKeep(Instance instance,
                                Literal newLit)
```

:   Test if an instance can be kept as a counter-instance,
    given a new literal.

    :   **Parameters:**: `instance` - The instance to test.: `newLit` - The new literal. **Returns:**: True if the instance is still a counter-instance.

---


### isIncludedIn

```
public abstract boolean isIncludedIn(Rule otherRule)
```

:   Test if this LiteralSet is included in a rule.

    :   **Parameters:**: `otherRule` - The rule to test. **Returns:**: True if this set of literals is included in the rule.

---


### toString

```
public abstract java.lang.String toString()
```

:   Gives a String representation for this set of literals.

    :   **Overrides:**: `toString` in class `java.lang.Object`


---


|  |  |  |
| --- | --- | --- |
| **Overview** \| **Package** \| **Class** \| **Tree** \| **Deprecated** \| **Index** \| **Help** \| **Weka's home** | |  |
| **PREV CLASS**   **NEXT CLASS** | **FRAMES**    **NO FRAMES**     **All Classes** |  |
| SUMMARY: NESTED \| FIELD \| CONSTR \| METHOD | DETAIL: FIELD \| CONSTR \| METHOD |  |


---
